# Ascending aorta graft pseudoaneurysm and aortobronchial fistula caused by a fractured sternal wire: a case report

**DOI:** 10.1186/s13019-021-01737-y

**Published:** 2021-12-07

**Authors:** Ahmad Ali Amirghofran, Elahe Nirooei, Mohammad Ali Ostovan

**Affiliations:** 1 Cardiac Surgery Department, Shiraz University of Medical Sciences, Shiraz, Iran; 2 Cardiovascular Research Center, Shiraz University of Medical Sciences, Shiraz, Iran; 3 Cardiology Department, Shiraz University of Medical Sciences, Shiraz, Iran

**Keywords:** Sternal wire, Pseudoaneurysm, Aortobronchial fistula, Aortic operation, Aneurysm, Cardiac, Mediastinum, Surgery/complications

## Abstract

**Background:**

Pseudoaneurysm of ascending aorta is a rare but serious complication of cardiovascular surgeries and it infrequently occurs in the normal prosthetic graft materials. We share our experience with an unusual case of ascending aorta Dacron graft pseudoaneurysm caused by a fractured sternal wire.

**Case presentation:**

A 34-year-old man, known case of Marfan syndrome, with history of two prior aortic surgeries for aneurysm of ascending aorta, arch and thoracoabdominal aorta, presented with hemoptysis. The hemoptysis originated from an aortobronchial fistula secondary to a huge ascending aorta Dacron graft pseudoaneurysm. The graft erosion and subsequent pseudoaneurysm was caused by a fractured sternal wire. Surgical repair of the pseudoaneurysm was performed successfully and a Gore-tex patch was placed behind the sternum over the graft to prevent further direct contact of the wire and the graft.

**Conclusion:**

Sternal wires can damage the adjacent vascular grafts and lead to fatal complications such as pseudoaneurysm formation. Thus, preventive measures such as using sternal bands and placing a covering layer between the sternal wires and aortic grafts are recommended in patients with dilated or replaced ascending aorta.

## Introduction

Pseudoaneurysm of ascending aorta is a rare but serious complication of cardiovascular surgeries. It is typically associated with aortotomy, aortic cannulation sites, anastomotic suture lines, and needle puncture sites, but it infrequently occurs in the normal prosthetic graft materials [1, 2]. This manuscript unfolds an unusual case of ascending aorta Dacron graft pseudoaneurysm caused by a fractured sternal wire.

## Case presentation

A 34-year-old man, known case of Marfan syndrome, presented with three episodes of non-massive hemoptysis. Seven years earlier, he had undergone Bentall operation and arch replacement with E-vita graft using frozen elephant trunk (FET) technique with a stent-graft in the descending aorta due to acute aortic dissection and aortic arch aneurysm. Six years later, he had returned with a thoraco-abdominal aortic aneurysm and a huge pseudoaneurysm around the distal part of the descending aorta. An extensive surgery with thoracoabdominal incision replacing all the thoracic and abdominal aorta by Dacron graft had been done, and the proximal part of the graft had been sutured to the distal end of the stent-graft.

**Fig. 1:**
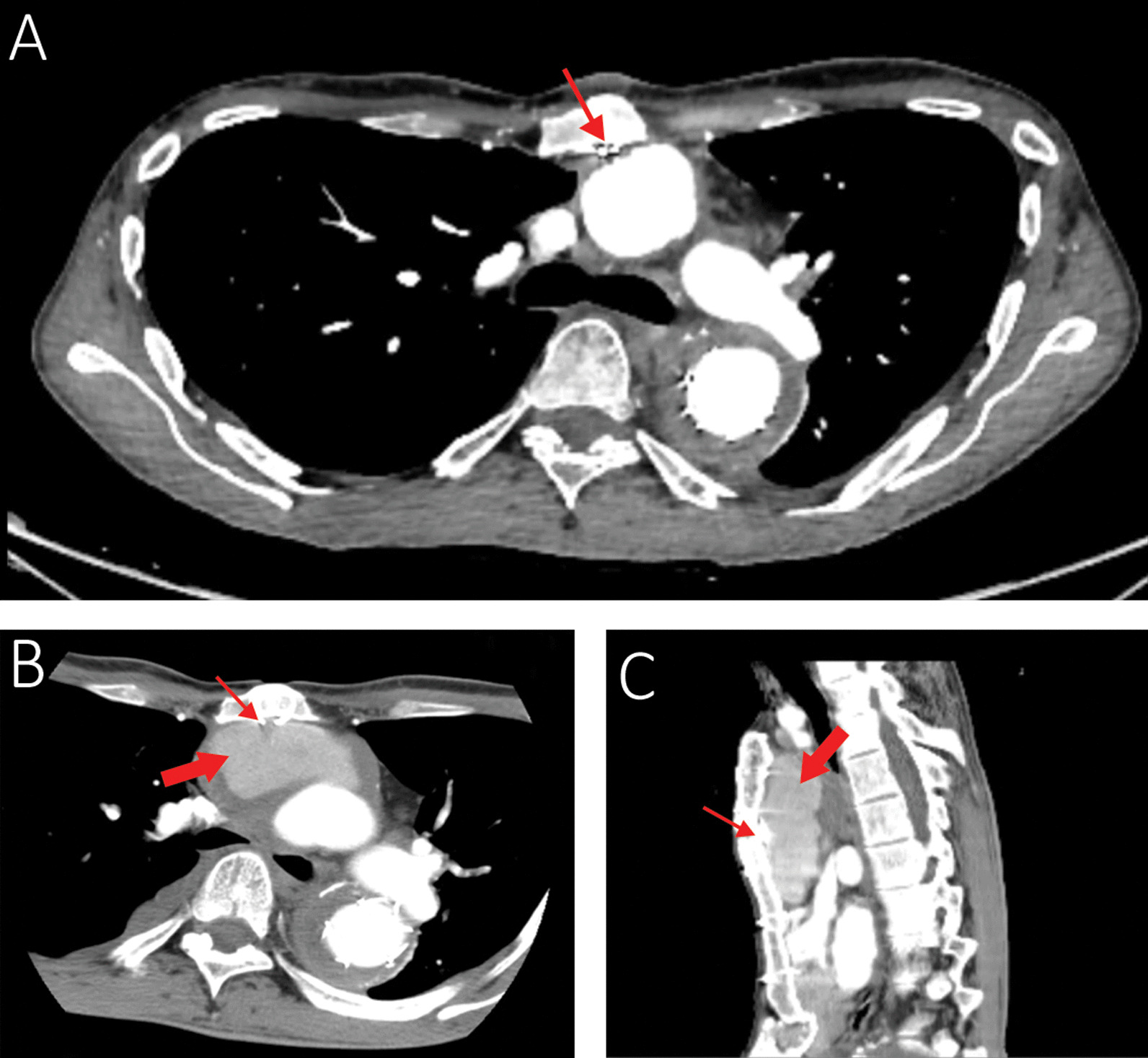
**A** The CTA performed six months before presenting the hemoptysis. Note the proximity of the sternal wire and the graft and the absence of any space between the sternum and the aortic graft. **B** and **C** The recent CTA (axial and lateral views): the thin arrow shows the fractured wire, and the thick arrow shows the pseudoaneurysm. Note the large space filled with a hematoma between the graft and the posterior border of the sternum

Finding out the cause of hemoptysis, he was admitted and a comprehensive evaluation including computed tomography angiography (CTA), bronchoscopy, and transesophageal echocardiography set out. The CTA showed a new collection, most probably a pseudoaneurysm sized 4 × 6 × 9 cm around the ascending aorta extending to the posterior border of the manubrium at the maximum diameter (Fig. 1B, C). Therefore, we focused on the ascending aorta as the source of bleeding into the mediastinum, which could have caused an aortobronchial fistula and ultimately the hemoptysis. The source images of a CTA belonging to 6 months earlier were reviewed. We noticed a sternal wire applied with the figure-of-eight technique in the first surgery in close contact with the Dacron graft, and it seemed to have penetrated the graft (Fig. 1A). We also spotted a fracture line on the wire without any displacement. Viewing a large space between the wire and the graft occupied by a pseudoaneurysm in the latest CTA corroborated the assumption that the graft erosion was established by the wire.

The episodes of hemoptysis decreased to only one in his admission course, but the patient progressed with a fever. Work-ups ruled out COVID19 infection but revealed a positive Enterobacter blood culture for which the patient received wide spectrum antibiotics for two weeks. Even though later cultures were negative and transesophageal echo did not indicate endocarditis, a low-grade fever was still running. The mediastinal hematoma and perhaps its connection to the respiratory system was presumed to be the main reason for the fever. Endovascular approach and stent grafting was excluded because of the possibility of the presence of an infection. So, our team decided on surgical treatment.

## Surgical technique

**Fig. 2:**
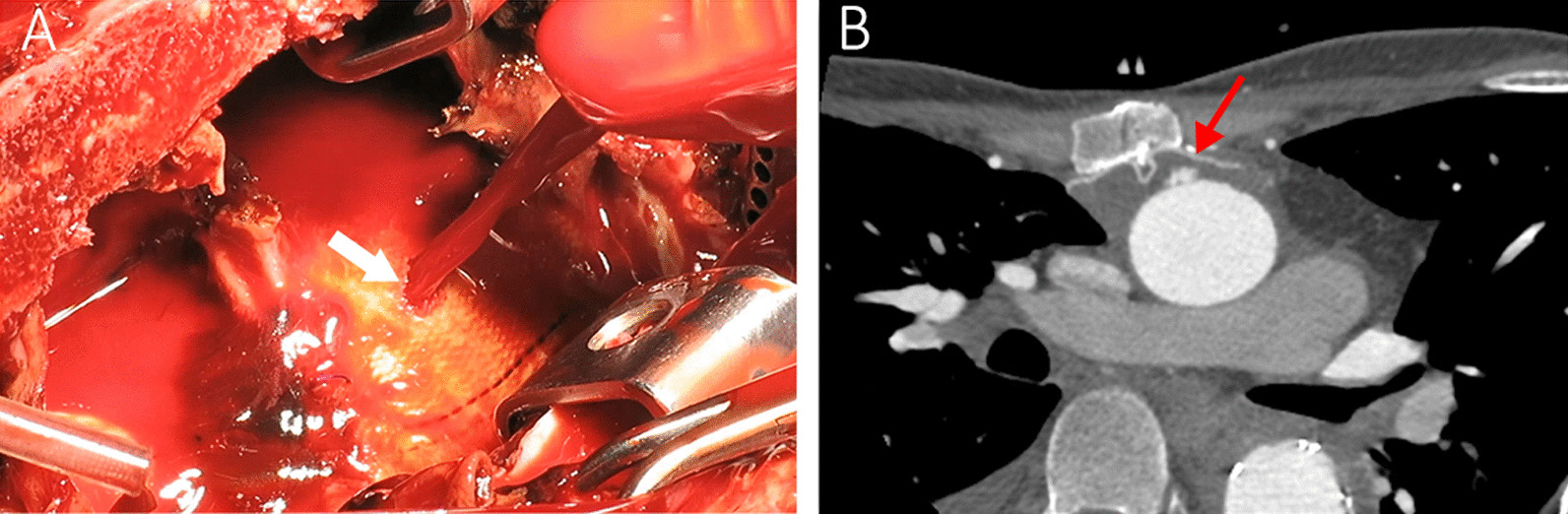
**A** The erosion hole on the Dacron graft and the outburst of blood shown by a white arrow. **B** The CTA performed three months after the repair. Note the Gore-tex patch placed between the sternum and the aortic graft

Since the lower half of the sternum had severe inward deviation due to pectus excavatum and the suspected graft perforation was at the level of the manubrium, minimally invasive upper reverse J sternotomy was selected. The femoral vessels were exposed and cannulated. The innominate artery was also exposed and controlled above the sternal notch in case there was any need for selective cerebral perfusion. Cardiopulmonary bypass was started, and the sternum was opened at a temperature of 32°. The blood outburst from the aorta was recovered by sump suction back into the circulation, and the bypass flow rate was decreased to minimize the bleeding. An approximately 5 × 8 mm hole was identified on the Dacron graft, finger controlled and then directly repaired by reinforced 3-0 Prolene suture (Fig. 2A). The hematoma components around the graft were evacuated. Based on our assumption that the aortobronchial fistula had been almost certainly sealed with clots, we did not manipulate the deeper parts around the trachea. A 0.4 mm Gore-tex patch was also placed behind the sternum over the graft to prevent further direct contact of the wire and the graft (Fig. 2B). The rest of the operation and the postoperative course passed smoothly and uneventfully, and the clot cultures were negative. No fever or hemoptysis was reported afterwards, and the 3-month follow-up CTA was satisfactory.

## Discussion and conclusions

Sternal wire fractures which occur after the median sternotomy are not rare radiologic findings. Although the strength induced by force under normal conditions is far below the ultimate tensile strength of the wire, the fracture of steel wires might occur after routine procedures. The mechanism is attributed to mechanical or chemical cracking secondary to bending and twisting or exposure to body fluids highly concentrated in chloride ions. Upper body activities and repetitive thorax movements such as respiration and coughing might also precipitate wire failures. Imperfect manufacturing and sterilization processes are other factors influencing the structure of wires [3, 4].

In our case, the incident of wire fracture early after the second surgery despite no infection or sternal dehiscence made us contemplate whether the force caused by the thoracoabdominal incision and rib retraction during the second surgery had affected the normal constitution and strength of the sternal wire.

Different types of intrathoracic injuries caused by the fracture and migration of wires have been reported such as right ventricle to aorta fistula [5], intravascular embolization (into the right lower pulmonary artery and aorta) [6, 7] and erosion into the right middle bronchus [3]. However, aortic graft-associated injuries are extremely rare. Up to our knowledge, only Kao et al. [1] reported a similar case of ascending aortic graft pseudoaneurysm caused by sternal wires. We also described our case because this is a subject that needs to be more highlighted. As mentioned, we did rewiring and used a Gore-tex patch between the sternal wires and the aortic graft as a preventive measure to eliminate their contact and minimize the possible injuries. However, in cases with dilated ascending aorta or aortic graft, using sternal bands instead of wires for the closure of the manubrium, which is adjacent to the aorta, would be the safest approach. Placing a covering layer from the surrounding tissue or a synthetic material between the sternum and the graft could reinforce stability.

## Data Availability

Data sharing is not applicable to this article as no datasets were generated or analyzed during the current study**.**

## Abbreviations

CTA: Computed tomography angiography
COVID-19: Corona virus disease of 2019
